# Editorial: Neuroplasticity in Rehabilitation

**DOI:** 10.3389/fresc.2022.916174

**Published:** 2022-05-25

**Authors:** Max O. Krucoff, Iahn Cajigas, Igor Lavrov

**Affiliations:** ^1^Department of Neurosurgery, Medical College of Wisconsin, Milwaukee, WI, United States; ^2^Department of Biomedical Engineering, Medical College of Wisconsin Graduate School of Biomedical Sciences and Marquette University, Milwaukee, WI, United States; ^3^Department of Neurosurgery, University of California, San Francisco, San Francisco, CA, United States; ^4^Department of Neurology, Mayo Clinic, Rochester, MI, United States

**Keywords:** neurorehabilitation, brain injury, CNS injury and repair, spinal cord injury, neuromodulation

We are thrilled to present *Neuroplasticity in Rehabilitation*, a Research Topic exploring how the nervous system changes in response to rehabilitative interventions. While the field of human neural interfacing began with the development of bypass devices (1, 2), or machines designed to circumvent neurological injury to execute an intended action, more recent discoveries enable us to study how such interventions directly modulate the nervous system and induce functional reorganization (3). Because neuroplasticity drives recovery (4), we hope such knowledge will one day translate to the ultimate goal of complete neurological healing.

The sheer number of modalities available for rehabilitation and the study of its effects on the human nervous system has rapidly expanded, and this Research Topic represents a sampling of active areas of investigation. Interventions presented here span classical rehabilitation therapies like stepping (Tansey et al.), botulinum neurotoxin injection (Vinehout et al.), and rapid-reciprocal leg training (Damiano et al.), to newer techniques like robotic-assisted therapies (Chen et al.; van Hedel et al.), biofeedback (van't Veld et al.), and exoskeleton-based walking with trans-spinal stimulation (Sutor et al.). Similarly, resultant neuroplasticity is measured with a range of classical functional outcome measures (Chen et al.) and reflex arc assessments (Tansey et al.; van't Veld et al.) to newer imaging-based surrogates of brain activation and connectivity such as functional magnetic resonance imaging (fMRI) (Vinehout et al.; Damiano et al.), diffusion tensor imaging (DTI) (Damiano et al.), and functional near-infrared spectroscopy (fNIRS). Finally, Proulx et al. round out our topic by providing a thoughtful narrative review on the importance of multimodal sensory feedback in neurorehabilitation strategies, making a case for the importance of intentionally incorporating brain multisensory integration areas during motor learning to improve motor outcomes in stroke.

Despite such technological progress, the path to clinical translation and widespread implementation of novel neurorehabilitation techniques remains long and arduous. As accessibility to advanced computational power has become more mainstream, technical limitations are no longer the major hurdle to offering efficacious therapies for central nervous system (CNS) repair; instead, it is our persistently incomplete understanding of human CNS organization, regeneration, and plasticity. Because the CNS is such a complex system that functions on physical scales ranging from microscopic/molecular to full human body circuitry and interacts on timescales ranging from near-instantaneous to a lifetime (3), obtaining a comprehensive understanding and fully accurate model seems nearly impossible.

However, even short of a complete understanding, incremental progress and discoveries are having tangible impacts. For example, we are now seeing unprecedented recovery from spinal cord injury (5–8), novel therapeutic options for stroke (9), neurostimulation techniques to expand surgeries into previously unresectable cortex (10), speech prostheses for those who cannot talk (11), and closed-loop neuromodulation techniques for movement disorders (12) and psychiatric illness (13). To continue advancing the field in a productive direction where patients can benefit along the way, cross-collaboration across niche and siloed expertise, as well as between academia, industry, researchers, and clinicians, will be critical. We hope it is clear from this Research Topic that incremental advances in neurorehabilitation can be meaningful, and that more impactful innovations come when many disciplines work together.

## Author Contributions

All authors listed have made a substantial, direct, and intellectual contribution to the work and approved it for publication.

## Conflict of Interest

The authors declare that the research was conducted in the absence of any commercial or financial relationships that could be construed as a potential conflict of interest.

## Publisher's Note

All claims expressed in this article are solely those of the authors and do not necessarily represent those of their affiliated organizations, or those of the publisher, the editors and the reviewers. Any product that may be evaluated in this article, or claim that may be made by its manufacturer, is not guaranteed or endorsed by the publisher.
